# Correlations between moral courage scores and social desirability scores among medical residents and fellows in Argentina

**DOI:** 10.3352/jeehp.2020.17.6

**Published:** 2020-02-18

**Authors:** Raúl Alfredo Borracci, Graciana Ciambrone, José María Alvarez Gallesio

**Affiliations:** 1 School of Medicine, Buenos Aires University, Buenos Aires, Argentina; 2 Medical Education Research Laboratory, Hospital Alemán, Buenos Aires, Argentina; Hallym University, Korea

**Keywords:** Moral Obligations, Courage, Medical Residencies, Psychometrics, Desirability, Social, Argentina

## Abstract

**Purpose:**

Moral courage refers to the conviction to take action on one’s ethical beliefs despite the risk of adverse consequences. This study aimed to evaluate correlations between social desirability scores and moral courage scores among medical residents and fellows, and to explore gender- and specialty-based differences in moral courage scores.

**Methods:**

In April 2018, the Moral Courage Scale for Physicians (MCSP), the Professional Moral Courage (PMC) scale and the Marlowe-Crowne scale to measure social desirability were administered to 87 medical residents from Hospital Alemán in Buenos Aires, Argentina.

**Results:**

The Cronbach α coefficients were 0.78, 0.74, and 0.81 for the Marlowe-Crowne, MCSP, and PMC scales, respectively. Correlation analysis showed that moral courage scores were weakly correlated with social desirability scores, while both moral courage scales were strongly correlated with each other. Physicians who were training in a surgical specialty showed lower moral courage scores than nonsurgical specialty trainees, and men from any specialty tended to have lower moral courage scores than women. Specifically, individuals training in surgical specialties ranked lower on assessments of the “multiple values,” “endurance of threats,” and “going beyond compliance” dimensions of the PMC scale. Men tended to rank lower than women on the “multiple values,” “moral goals,” and “endurance of threats” dimensions.

**Conclusion:**

There was a poor correlation between 2 validated moral courage scores and social desirability scores among medical residents and fellows in Argentina. Conversely, both moral courage tools showed a close correlation and concordance, suggesting that these scales are reasonably interchangeable.

## Introduction

### Background/rationale

Moral courage refers to the conviction to take action on one’s ethical beliefs despite the risk of consequences, and such courage is critical to physicians’ commitment to act in the best interest of patients. In daily clinical practice, situations requiring moral courage include, for instance, delivering care to an infectious patient, meeting a displeased patient or an angry family member, addressing an incompetent colleague, disclosing a medical error, and raising concerns about unethical or unsafe practices [1,2]. A practical scale for measuring moral courage in patient care was recently proposed by Martínez et al. [1]. This 9-item scale is known as the Moral Courage Scale for Physicians (MCSP), and it is based on previously described relevant dimensions as follows: the predisposition to behave ethically and work toward what is right (known as moral agency); the ability to draw on multiple sets of values in ethical decision making (multiple values); the conviction to do and the tendency to reflect on what is right or just (endurance of threats); and a virtuous motivation to do what is right, as opposed to being motivated by self-interest (moral goals) [1,3]. Four validated scales to measure courage have been previously developed [4], of which 2—Hannah’s scale [5] and the Professional Moral Courage (PMC) scale—measure moral courage [2]; however, neither focus on clinical medicine. Sekerka et al. [3] developed the PMC scale to study moral courage among military personnel who work in morally complex and hierarchical settings where exemplary behavior is critical.

Socially desirable responding refers to individuals’ tendency to present a favorable image of themselves by answering in conformity to socially acceptable values to avoid criticism or gain social approval. It is most likely to occur in response to socially sensitive questions, and response bias may affect the validity of the questionnaire. Several scales have been developed to measure the influence of potential socially desirable responses, of which the Marlowe-Crowne Social Desirability scale is one of the most widely used. This scale contains true or false statements that influence the individual to respond in a manner that conforms to social expectations regarding behaviors, attitudes, and beliefs. The effect of social desirability can be observed in individuals who tend to seek approval from others or who may distort their right behavior to make a good impression. It is commonly described as a measure of a person’s need for approval, and individuals with a high need for approval would tend to score high on the social desirability scale.

### Objectives

We hypothesized that there would be a weak correlation between the moral courage scales and social desirability measurements (the null hypothesis for correlations between the MCSP/PMC and Marlow-Crowne scales). Conversely, a strong correlation was expected between both moral courage scores (alternative hypothesis for a correlation between MCSP and PMC scales). The primary objective of this study was to evaluate the correlation between social desirability scores and moral courage assessed with the MCSP and PMC tools among medical residents and fellows. Since in the original study of Martínez et al. [1], multivariate regression analysis demonstrated gender- and specialty-based differences in MCSP scores, we proposed to explore these associations among our physicians-in-training as a secondary objective.

## Methods

### Ethics statement

Participants were assured that the completed questionnaire would be anonymous and confidential. After being informed of the general purpose of the study, respondents voluntarily participated in the survey and consent was implied by survey completion. The protocol was evaluated and approved by the Institutional Review Board of Hospital Alemán in Buenos Aires, Argentina (reference DH2018-19).

### Study design

This was a survey questionnaire-based cross-sectional study. It consisted of 2 parts: (1) testing the validity and reliability of 2 Spanish-language versions of moral courage measurement tools, and (2) analyzing the correlations between moral courage scales and a social desirability measurement.

### Participants

In April 2018, a total of 108 medical residents and fellows from an academic community hospital associated with Buenos Aires University were invited to participate in a study conducted at the School of Medicine of Buenos Aires. All medical residents and fellows (first-year and above) were included in the survey. Eighty-five physicians-in-training (78.7%) were enrolled in a residency program and the rest were enrolled in a fellowship program. Among the participants, 56% (n=60) were training in a nonsurgical program, and the rest were training in a surgical specialty. The first-year residents had spent at least 10 months in the program when they were surveyed. Participants completed the questionnaire voluntarily and anonymously.

### Measurement

A Spanish version of the MCSP was administered in a hard copy alongside the Spanish version of the PMC scale 2 described by Sekerka et al. [3]. As reported by the authors who developed the scale, 3 items (numbers 9, 12, and 15) were omitted from the questionnaire, since respondents tended to answer the negatively phrased items inconsistently, apparently not realizing the inverted wording. Since the MCSP was partially derived and adapted from the PMC scale, the inclusion of this latter scale in the current study was intended to serve as a measure of concurrent validity. The exact wording of items in the MCSP and PMC scales is summarized in the left column of Table 1; this column also contains the list of the 5 moral courage themes or dimensions of the PMC scale. Two researchers (RAB, GC) independently translated the original English versions of the MCSP and the PMC scale into Argentinian Spanish (Supplement 1). They discussed and resolved differences in their translations and reached a consensus on the best initial wording. The results were back-translated by an independent bilingual translator who was unaware of the original English versions. The back-translations conveyed the original meaning, and for many statements, were identical to the original wording. Items were left in the same order as in the originals, and the same 7-point Likert scale response structure was used for both scales. Finally, to control whether the answers to the items on the moral courage scales could be biased by a tendency to respond in a socially desirable way, a previously validated Spanish version of the original Marlowe-Crowne scale was simultaneously administered as a measure of social desirability [6].

### Setting

All participants responded to items from the MCSP, the PMC scale, and the Marlowe-Crowne scale. In addition, information about age and gender was required to complete the form. Respondents took less than 25 minutes to answer the 52 questions on the three questionnaires (question #1 [“I am determined to do the right thing”] and question #2 [“Others can rely on me to exemplify moral behavior”] from the PMC scale were eliminated to avoid duplication with the equivalent questions included in the MCSP). There was no economic incentive for completing the questionnaires.

### Statistical analysis

A summary score for MCSP was computed using the following formula: scale score=(average score across all scale items–1)×(100/6). Thus, the summary scores for the scale ranged from 0 (worst) to 100 (best). For each dimension of moral courage in the PMC scale, items were averaged to yield a measure of that dimension, and the total sum of the 5 original dimensions was recorded as the final PMC score. After reverse-coding the negatively worded items, the total sum of the 33 items from the Marlowe-Crowne scale was used as a measure of social desirability. The internal reliability of the 3 scales was assessed with the Cronbach α coefficient, and values >0.70 were considered acceptable. Parametric and nonparametric correlations among the 3 scales were assessed with Pearson r and Spearman rho coefficients. In addition, the effect size was reported based on current guidelines, and correlations of 0.10, 0.20, and 0.30 were considered to be small, moderate, and large, respectively [7]. The value of explained variance as a percentage was obtained by squaring the Pearson correlation coefficient (r² or determination coefficient). The explained variance (also called explained variation) is the proportion of the variance in the dependent variable that is explained or predicted by the independent variable. The presence of outliers was analyzed by computing bagplots for bivariate data sets [8]. The Bland-Altman concordance method and the intraclass correlation coefficient were used to assess agreement between the MCSP and the PMC scale employing Epidat ver. 4.1 (OPS-OMS, Santiago de Compostela, Spain). The Kolmogorov-Smirnov goodness-of-fit test was used to analyze the normality of the distribution of data, and univariate comparison of metric variables was performed using the Student t-test assuming Gaussian distributions. Continuous variables were expressed as mean and standard deviation (SD). Since multiple statistical comparisons were made between surgical and nonsurgical specialties, and between males and females, family-wise error rates were calculated and corrected with the Holm-Bonferroni method to adjust each P-value. Based on this method, a threshold of P=0.006 was adopted for the MCSP and a threshold of P=0.010 for the PMC scale, for both specialty- and gender-based comparisons. Univariate comparison of dichotomous variables was performed using the chi-square (χ²) test. Statistical analyses were performed with SPSS for Windows ver. 17.0 (SPSS Inc., Chicago, IL, USA) and a 2-tailed P-value ≤0.05 was considered to indicate statistical significance. When reporting the characteristics of an existing scale, the analysis should confirm that the structure was the same despite translation. Since the dimensional structures of the MCSP and the PMC scale have already been established, it was appropriate to conduct confirmatory factor analysis (CFA), instead of principal component analysis. CFA investigates how the data fit into a predetermined and constructed model by presenting the relationship between the data in the model and the estimation of errors. LISREL ver. 9.20 (Scientific Software International Inc., Skokie, IL, USA) was used to test the structure of the Spanish versions of the MCSP and the PMC scale by CFA. Model data fit was assessed using the maximum likelihood ratio χ² test, root mean square error of approximation (RMSEA), comparative fit index (CFI), and standardized root mean square residual (SRMR). A nonsignificant χ² (P>0.05), RMSEA and SRMR <0.08, and CFI >0.9 were considered to be indicators of adequate model fit.

## Results

### Participants

The questionnaires were completed by 87 (81%) of the 108 eligible medical residents and fellows. The average age of the respondents was 29 (SD=2.3) years, and gender, specialties, and position did not differ significantly from those of the total population surveyed.

### Reliability and construct validity

The Cronbach α coefficients were 0.78, 0.74, and 0.81 for the Marlowe-Crowne, MCSP, and PMC scales, respectively. CFA yielded the following goodness-of-fit statistics for the MCSP: χ²=13.3 (P=0.021), RMSEA=0.138 (90% confidence interval [CI], 0.049–0.230), CFI=0.920, and SRMR=0.071. The same indexes for the PMC scale were χ²=7.3 (P=0.199), RMSEA=0.073 (90% CI, 0.00–0.178), CFI=0.981, and SRMR=0.043. An adequate fit was observed for the PMC scale according to all indexes, but only a partial fit for the MCSP based on the CFI and SRMR statistics.

### Correlations between scales

**Fig. 1.**
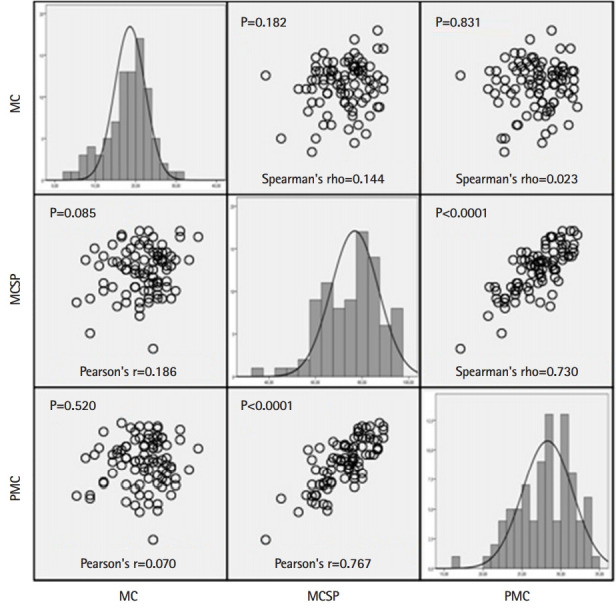
Matrix scatterplot showing parametric and nonparametric correlations between the MC, the MCSP, and the PMC. The diagonal represents the distribution of values for each individual scale. MC, Marlowe-Crowne Social Desirability scale; MCSP, Moral Courage Scale for Physicians; PMC, Professional Moral Courage scale.

**Fig. 2.**
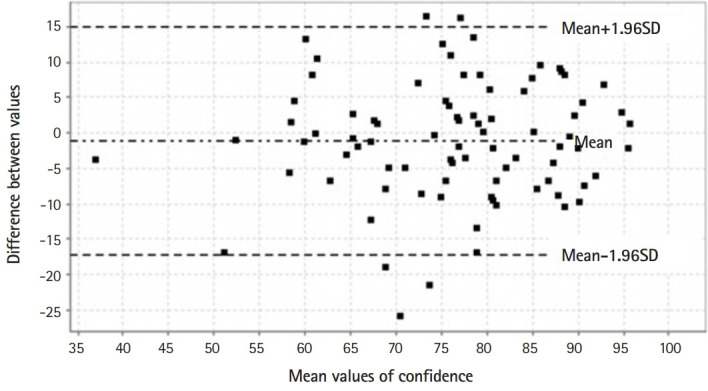
Bland-Altman plot of the difference between the MCSP and the PMC scale in the overall cohort. For comparative purposes, the PMC score was computed using the same formula proposed for the MCSP. The average bias between the MCSP and the PMC scale was -1.16 (95% confidence interval, -2.93 to 0.60). Since this confidence interval includes zero, it can be considered that both scales show overall concordance for assessing moral courage. MCSP, Moral Courage Scale for Physicians; PMC, Professional Moral Courage; SD, standard deviation.

Fig. 1 summarizes the distribution of values for each individual scale and the matrix scatterplot of the parametric and nonparametric correlations between the Marlowe-Crowne scale and both moral courage scales (MCSP and PMC). The correlation analysis showed that moral courage scores had a poor correlation with social desirability scores, with a small effect size. Hence, the responses to the MCSP and the PMC scale seemed to be independent of socially acceptable behavior as assessed by the Marlowe-Crowne scale. Conversely, there was a strong correlation and large effect size between both moral courage scales. Fig. 2 represents the Bland-Altman plot of the difference between the MCSP and the PMC scale, while the intraclass correlation coefficient between both moral courage scales was 0.768 (95% CI, 0.666–0.842). Biases between the MCSP and the PMC scale were obtained from Bland-Altman analysis. The intraclass correlation level demonstrated overall concordance between the 2 scales when assessing moral courage. Moderate correlations were found between age and MCSP (r=0.203, P=0.060) and PMC (r=0.179, P=0.098) scores.

### Specialty- and gender-based differences

Table 1 contains the mean Likert scale scores for the moral courage items and themes of the MCSP and the PMC scale, respectively, separated by nonsurgical and surgical specialties. Overall, individuals training in a surgical specialty ranked lower on the MCSP on the “multiple values” (question 6) (P=0.017) and “endurance of threats” (question 8) (P=0.029) dimensions. The same results were found in this group when assessing “multiple values” (theme 2) (P=0.027), “endurance of threats” (theme 3) (P=0.028), and “going beyond compliance” (theme 4) (P=0.039) on the PMC scale. Differences in moral courage scores were also observed according to gender (Table 2). Men tended to rank lower than women on the “multiple values” (question 6) (P<0.0001) and “moral goals” (question 7) (P=0.005) dimensions of the MCSP, and on “endurance of threats” (theme 3) (P=0.032) and “moral goals” (theme 5) (P=0.041) on the PMC scale. However, when the Holm-Bonferroni correction was applied to address family-wise error rates, no statistically significant difference remained when comparing specialties; and men ranked significantly lower only on the “multiple values” and “moral goals” dimensions of the MCSP. The mean social desirability score based on the Marlowe-Crowne scale was 17.7 (SD=5.4) for the total cohort, 18.0 (SD=5.8) and 17.4 (SD=5.0) for women and men, respectively (P=0.624), and 17.8 (SD=5.9) and 17.5 (SD=4.5) for nonsurgical and surgical specialties, respectively (P=0.801).

## Discussion

### Key results

Since MCSP and PMC scores were poorly correlated with Marlowe-Crowne scale scores, it was concluded that responses to moral courage features were not associated with social desirability scores. Thus, the Marlowe-Crowne scale explained only 3% of variance in MCSP and less than 1% of variance in the PMC scale. In contrast and as expected, the MCSP was positively correlated with the other moral courage scale (PMC). These findings provide additional evidence for the validity of the MCSP as a measure of moral courage for physicians-in-training in the context of patient care. Moreover, although the original PMC scale was not specifically developed for the medical field, it was closely correlated with the MCSP. It showed adequate concordance, implying that the PMC scale may be a reasonable alternative for assessing moral courage among physicians.

### Comparison with previous studies

Previous reports showed that fewer than 1% of questionnaire-based studies used a social desirability scale to detect or control for desirability bias, and half of those using such a scale found that socially desirable responses influenced their results [9]. Furthermore, a recent systematic review including 35 studies highlighted some limitations in the use of social desirability scales in clinical psychology research [10]. Socially desirable responding was not previously assessed in the original MCSP, and to our knowledge, no external validation of this scale has been done until now.

On both moral courage scales, physicians-to-be who were being trained in a surgical specialty showed lower scores than nonsurgical specialty trainees on 3 core features of courage scores, including the ability to draw on and weigh multiple sets of values in ethical decision-making (the “multiple values” theme), the conviction to do what is right despite perceived or real threats to one’s self (the “endurance of threats” theme), and the tendency to consider more than compliance-based actions to do what is right (the “going beyond compliance” theme) [1,3]. Similarly, male trainees from all specialties tended to have lower moral courage scores than female trainees on the “multiple values” and “endurance of threats” themes, as well as on virtuous motivations to do what is right, as opposed to being motivated by self-interest for praise or reward (the “moral goals” theme). These findings may raise some concerns regarding the scarcity of some moral courage features among men and surgical specialty trainees, as compared with their counterparts. However, given the small sample size and the fact that this study was conducted at a single academic medical center, the findings of gender- and specialty-based differences should be considered only as a non-obvious result, since it was not possible to perform a multivariate analysis to assess potential confounders such as personality traits, tolerance of uncertainty, and religious education.

There is some empirical evidence that Latin Americans tend to have significantly higher social desirability scores than Europeans or Americans. Nevertheless, in this study, the mean score on the Marlowe-Crowne scale was lower than the values found in a Spanish population [11] and even in low-and middle-income countries of Africa, corrected for the 28-item version of the scale [12]. Regarding MCSP scoring, residents and fellows surveyed had lower mean scores than those observed by the researchers who developed the scale for all core features of moral courage, except for the “moral goals” dimension [1].

### Limitations

This study has several limitations. The survey was only administered to physicians-in-training at a single academic hospital; hence, the generalizability of these findings may be limited in other settings. Although the reliability of the translation could be strengthened by pretesting the items with a sample from the target population and using cognitive response interviews to ensure that the intended meaning of the items was conveyed, we did not use these tools in the current study. Since it is possible that Latin Americans score higher than Americans on social desirability scales, and the current survey found that our physicians ranked lower in most dimensions of moral courage measures than the participants in the original MCSP study [1], the potential correlations between the Marlowe-Crowne scale and moral courage scales might have been biased. Finally, although CFA showed good fit of the PMC scale, some inconsistencies among indexes arose in the MCSP model fitting. A possible explanation for these inconsistencies is that CFA needs a 20:1 ratio of the sample size to the number of free parameters in the model; hence, the low sample size of the present study may have impacted certain model fit indexes.

### Conclusions

Moral courage measurements based on self-reported behaviors may be not correlated with social desirability scores. In the current study, 2 validated moral courage scales showed poor correlations with a simultaneously administered measure of social desirability, ruling out a significant association between social desirability scores and moral courage scores among residents and fellows. Conversely, both moral courage tools showed a close correlation and high concordance, implying that these scales are reasonably interchangeable. The specialty- and gender-based differences in moral courage scores observed in our population may reflect suboptimal behaviors of certain groups when facing ethical and moral challenges in daily patient care. Assessing and cultivating moral courage should be considered as an institutional and educational priority. Future research should explore observed, rather than self-reported, moral courage in particular healthcare situations, such as delivering care to an infectious patient, or addressing an incompetent or impaired colleague. Furthermore, the relationships between moral courage and other related measures (e.g., bravery, burnout, and moral distress) could be studied, and it may also be fruitful to examine the effects of experience and targeted interventions on changes in moral courage scores over time.

## Figures and Tables

**Table 1.** Mean Likert-scale scores of the MCSP and the PMC scale, divided by nonsurgical and surgical specialties

	Overall	Nonsurgical specialties	Surgical specialties	P-value
MCSP items				
1. I do what is right for my patients, even if I experience opposing social pressures (e.g., opposition from senior members of the healthcare team, medical guidelines, etc.)	5.03±1.39	5.07±1.30	4.97±1.55	0.949
2. I use a guiding set of principles from my profession to help determine the right thing to do for my patients.	5.70±1.34	5.87±1.21	5.42±1.50	0.230
3. My patients and colleagues can rely on me to exemplify moral behavior.	5.78±1.04	5.80±1.02	5.76±1.09	0.890
4. I do what is right for my patients because it is the ethical thing to do.	6.20±0.97	6.20±0.98	6.18±0.98	0.962
5. I go above and beyond what is required to do what is right for my patients.	4.74±1.48	4.80±1.45	4.64±1.56	0.648
6. When faced with ethical dilemmas in patient care, I consider how both my professional values and my personal values apply to the situation before making decisions.	5.78±1.18	5.96±1.16	5.48±1.15	0.017
7. When I do the right thing for my patients, my motives are pure.	5.93±1.31	6.04±1.13	5.76±1.56	0.418
8. I do what is right for my patients, even if it puts me at risk (e.g., legal risk, risk to reputation, etc.).	4.16±1.88	4.56±1.72	3.52±1.97	0.029
9. I am determined to do the right thing for my patients.	6.49±0.87	6.52±0.84	6.45±0.94	0.647
PMC items				
Theme 1: moral agency	6.06±0.83	6.12±0.73	5.97±0.96	0.430
1. I am determined to do the right thing.				
2.Others can rely on me to exemplify moral behavior.				
3. Engaging in principled action is an ongoing pursuit for me.				
Theme 2: multiple values	4.92±1.09	5.09±1.09	4.66±1.06	0.027
4. I draw on my personal values to help determine what is right.				
5. I draw on the values of those around me to help determine what is right.				
6. I draw on my professional values to help determine what is right.				
Theme 3: endurance of threats	5.63±1.11	5.84±0.97	5.27±1.24	0.028
7. I hold my ground on moral matters, even if there are opposing social pressures.				
8. I act morally even if it puts me in an uncomfortable position with my superiors.				
Theme 4: going beyond compliance	5.71±0.91	5.90±0.85	5.41±0.95	0.039
9. I consider more than rules and regulations in deciding what is right.				
10. I proactively aspire to behave morally.				
Theme 5: moral goals	5.70±1.05	5.87±0.84	5.42±0.28	0.104
11.When I act morally, my motives are virtuous.				
12. I act morally because it is the right thing to do.				

Values are presented as mean±standard deviation. MCSP, Moral Courage Scale for Physicians; PMC, Professional Moral Courage.

**Table 2.** Mean Likert-scale scores of the MCSP and the PMC scale, divided by gender

	Female	Male	P-value
MCSP items			
1. I do what is right for my patients, even if I experience opposing social pressures (e.g., opposition from senior members of the healthcare team, medical guidelines, etc.)	5.003±1.31	5.07±1.49	0.766
2. I use a guiding set of principles from my profession to help determine the right thing to do for my patients.	5.76±1.52	5.64±1.12	0.277
3. My patients and colleagues can rely on me to exemplify moral behavior.	5.89±0.96	5.67±1.12	0.380
4. I do what is right for my patients because it is the ethical thing to do.	6.31±0.85	6.07±1.09	0.402
5. I go above and beyond what is required to do what is right for my patients.	4.87±1.41	4.60±1.56	0.383
6. When faced with ethical dilemmas in patient care, I consider how both my professional values and my personal values apply to the situation before making decisions.	6.16±1.17	5.38±1.06	0.000
7. When I do the right thing for my patients, my motives are pure.	6.31±1.00	5.52±1.49	0.005
8. I do what is right for my patients, even if it puts me at risk (e.g., legal risk, risk to reputation, etc.).	4.29±1.77	4.02±2.01	0.611
9. I am determined to do the right thing for my patients.	6.62±0.75	6.36±0.98	0.162
PMC items			
Theme 1: moral agency	6.13±0.82	5.99±0.83	0.424
1. I am determined to do the right thing.			
2. Others can rely on me to exemplify moral behavior.			
3. Engaging in principled action is an ongoing pursuit for me.			
Theme 2: multiple values	5.06±0.96	4.78±1.21	0.437
4. I draw on my personal values to help determine what is right.			
5. I draw on the values of those around me to help determine what is right.			
6. I draw on my professional values to help determine what is right.			
Theme 3: endurance of threats	5.88±0.98	5.36±1.19	0.032
7. I hold my ground on moral matters, even if there are opposing social pressures.			
8. I act morally even if it puts me in an uncomfortable position with my superiors.			
Theme 4: going beyond compliance	5.89±0.82	5.52±0.98	0.058
9. I consider more than rules and regulations in deciding what is right.			
10. I proactively aspire to behave morally.			
Theme 5: moral goals	5.87±1.08	5.52±0.99	0.041
11. When I act morally, my motives are virtuous.			
12. I act morally because it is the right thing to do.			

Values are presented as mean±standard deviation. MCSP, Moral Courage Scale for Physicians; PMC, Professional Moral Courage.

## Supplementary materials

Data files are available from https://doi.org/10.7910/DVN/0Q3F6Q

Supplement 1. Spanish version of the Moral Courage Scale for Physicians and the Professional Moral Courage scale.

Supplement 2. Audio recording of the abstract.
