# Supplementary material for: Correlations between moral courage scores and social desirability scores among medical residents and fellows in Argentina
Source: J Educ Eval Health Prof. 2020 Feb 18;17:6. doi: 10.3352/jeehp.2020.17.6 (PMC7364024; doi:10.3352/jeehp.2020.17.6)
Supplement: Supplementary file 2 — Supplement 1. Spanish version of the Moral Courage Scale for Physicians and the Professional Moral Courage scale. [file jeehp-17-06-suppl.pdf]

Supplement 1. Spanish version of the Moral Courage Scale for Physicians and the Professional Moral Courage scale

Spanish version of the Moral Courage Scale for Physicians

1. Hago lo que es correcto para mis pacientes, incluso si experimento presiones sociales contrarias (por ejemplo, la oposición de colegas mayores del equipo de atención médica, las pautas de las guías de práctica clínica, etc.)
2. Utilizo un conjunto guía de principios de mi profesión para ayudar a determinar lo que es correcto hacer para mis pacientes.
3. Mis pacientes y colegas pueden confiar en mí para ejemplificar el comportamiento moral.
4. Hago lo que es correcto para mis pacientes porque es lo ético que se debe hacer.
5. Voy más allá de lo necesario para hacer lo que es correcto para mis pacientes.
6. Cuando me enfrento a dilemas éticos en la atención del paciente, considero como aplicar mis valores profesionales y personales a la situación antes de tomar decisiones.
7. Cuando hago lo correcto para mis pacientes, mis motivos son puros.
8. Hago lo que es correcto para mis pacientes, incluso si ello me pone en riesgo (por ejemplo, riesgo legal, riesgo a la reputación, etc.).
9. Estoy decidido a hacer lo correcto para mis pacientes.

Spanish version of the Professional Moral Courage scale

Tema 1: Mediación Moral

1. Estoy decidido a hacer lo correcto.
2. Otros pueden confiar en mí para ejemplificar el comportamiento moral.
3. Busco constantemente la acción basada en principios.

Tema 2: Valores Múltiples

4. Me baso en mis valores personales para determinar lo que es correcto.
5. Me baso en los valores de quienes me rodean para determinar lo que es correcto.
6. Me baso en mis valores profesionales para determinar lo que es correcto.

Tema 3: Resistencia de las amenazas

7. Sostengo mis ideas en asuntos morales, aunque haya presiones sociales contrarias.
8. Actúo moralmente, aunque ello me ponga en una posición incómoda con mis superiores.

Tema 4: Más allá del cumplimiento

10. Considero otras cosas, aparte de reglas y regulaciones para decidir lo que es correcto.
11. Aspiro en forma proactiva a comportarme moralmente.

Tema 5: Metas Morales

13. Cuando actúo moralmente, mis motivos son virtuosos.
14. Actúo moralmente porque es lo correcto.

Note: As reported by the authors who developed the scale, 3 items (number 9, 12 and 15) were omitted from the questionnaire, since respondents tended to answer the negatively phrased items inconsistently, apparently not realizing the inverted wording.
